# Supplementary figures and images for: Discovering novel therapeutic VHHs for emerging viruses: perspectives from VEEV selection strategies
Source: Front Immunol. 2026 Mar 31;17:1806908. doi: 10.3389/fimmu.2026.1806908 (PMC13076302; doi:10.3389/fimmu.2026.1806908)

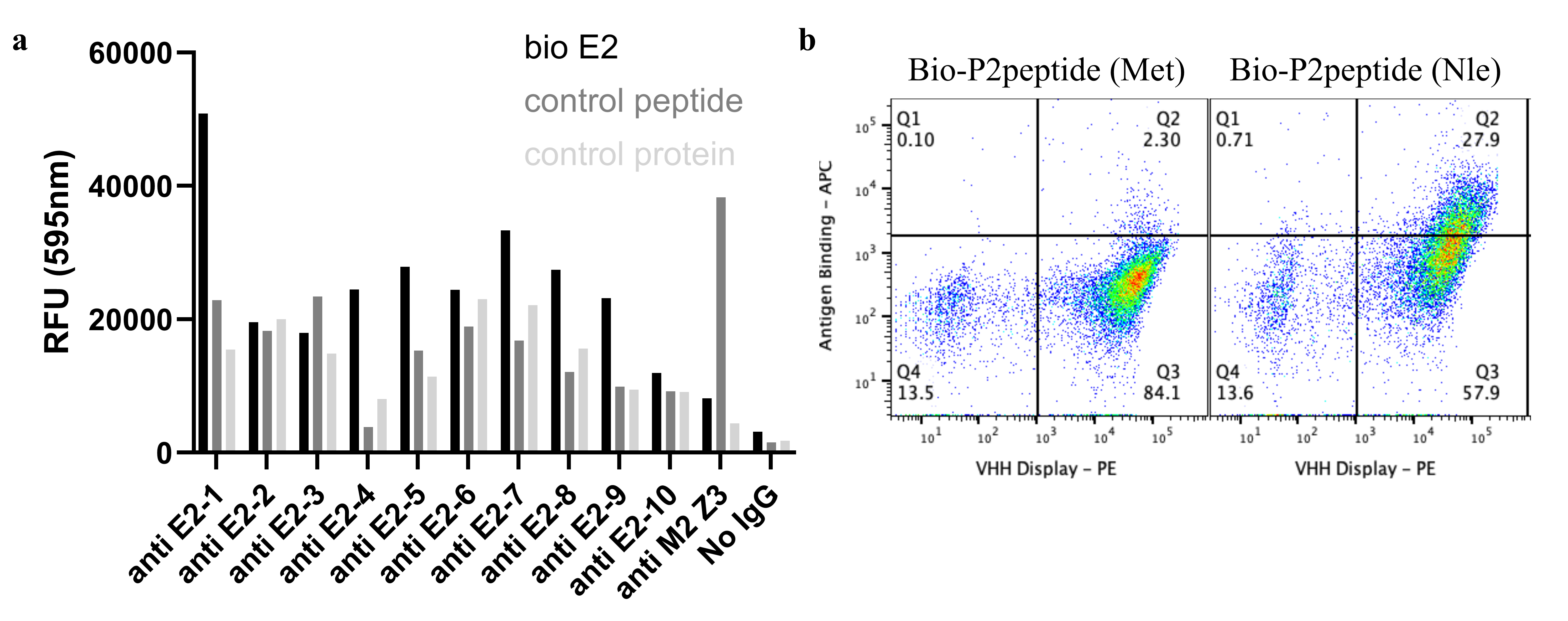

Supplement: Supplementary file 1 [file Image1.tif]

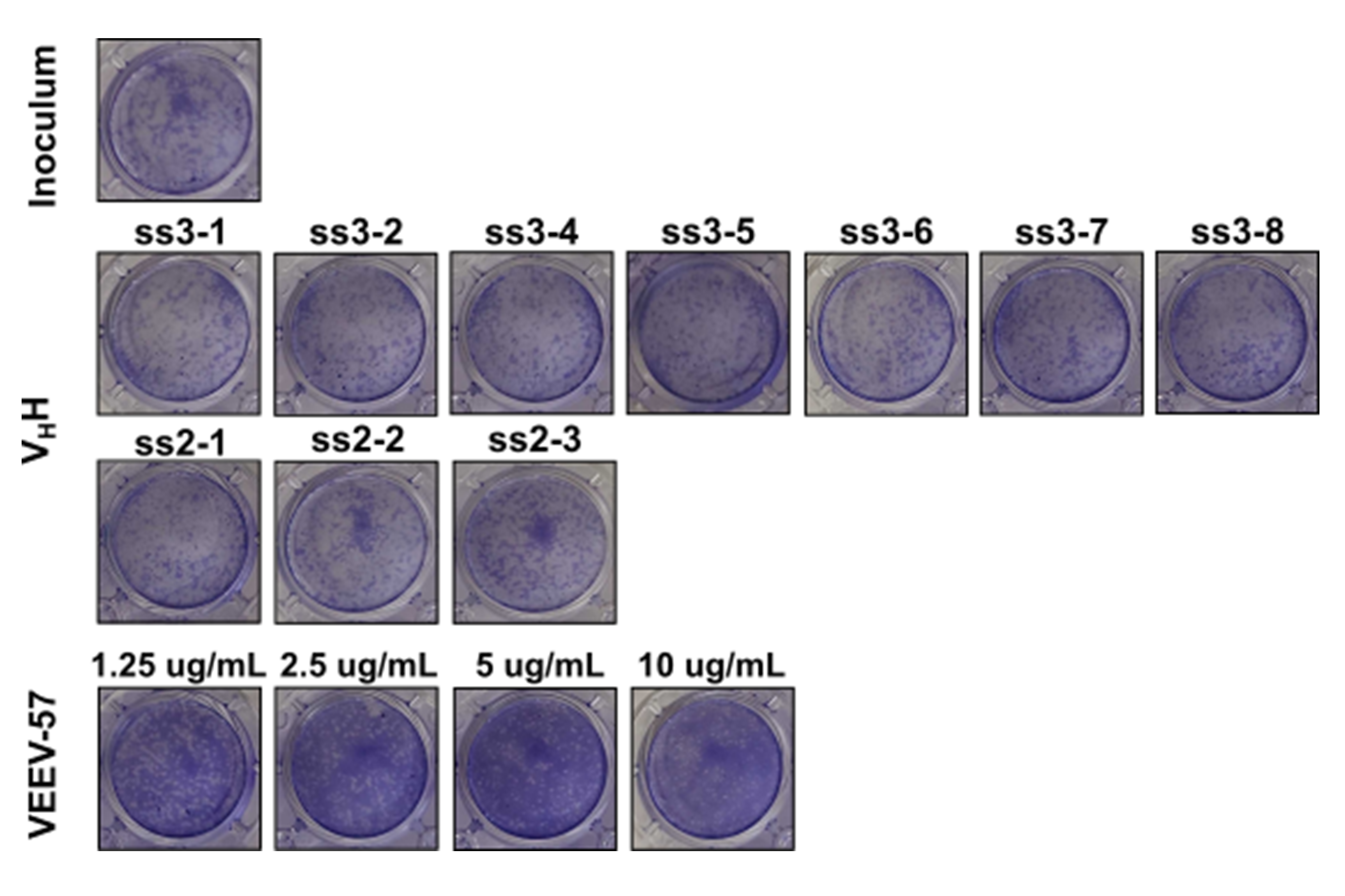

Supplement: Supplementary file 2 [file Image2.tif]

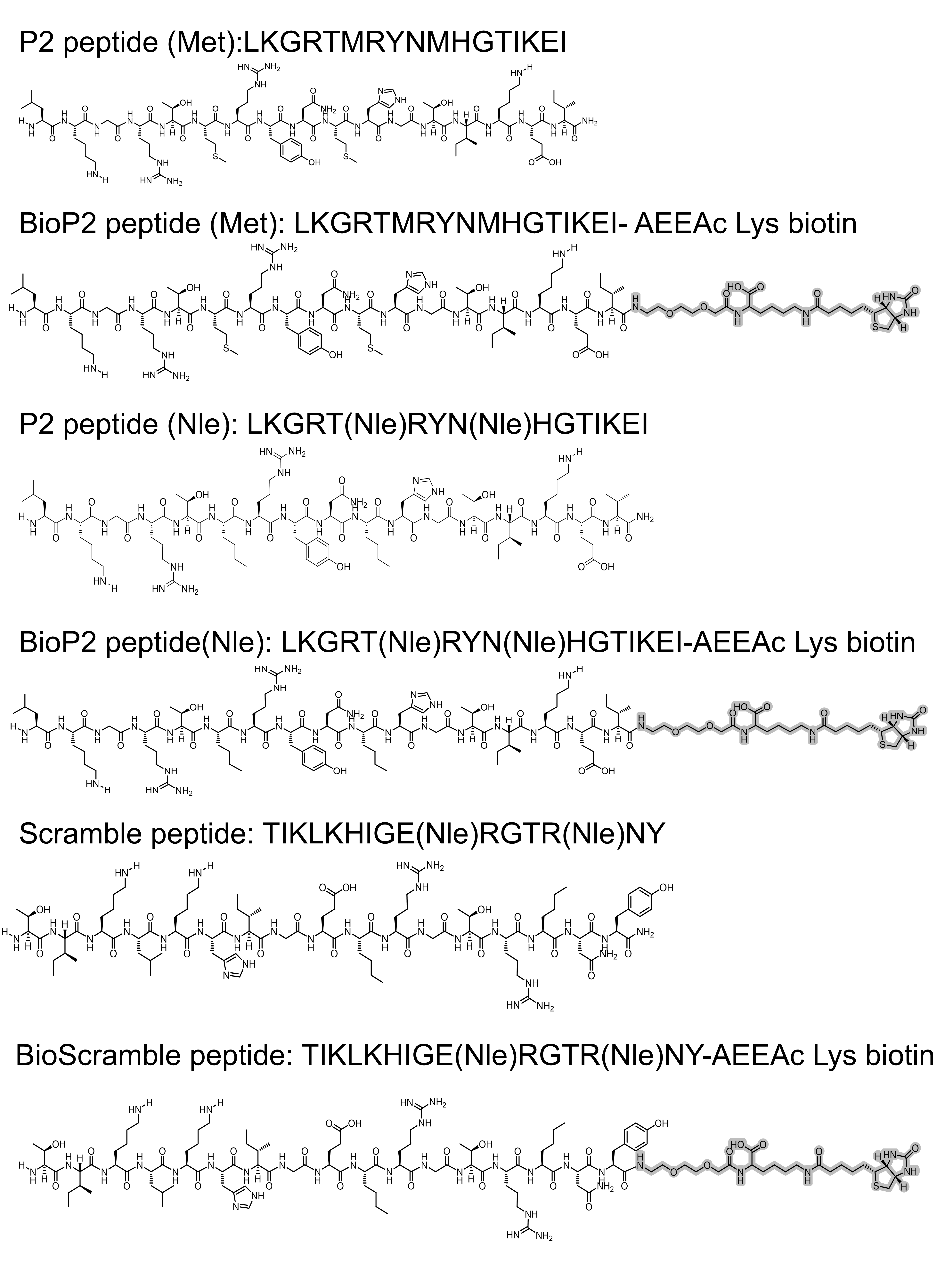

Supplement: Supplementary file 3 [file Image3.tif]

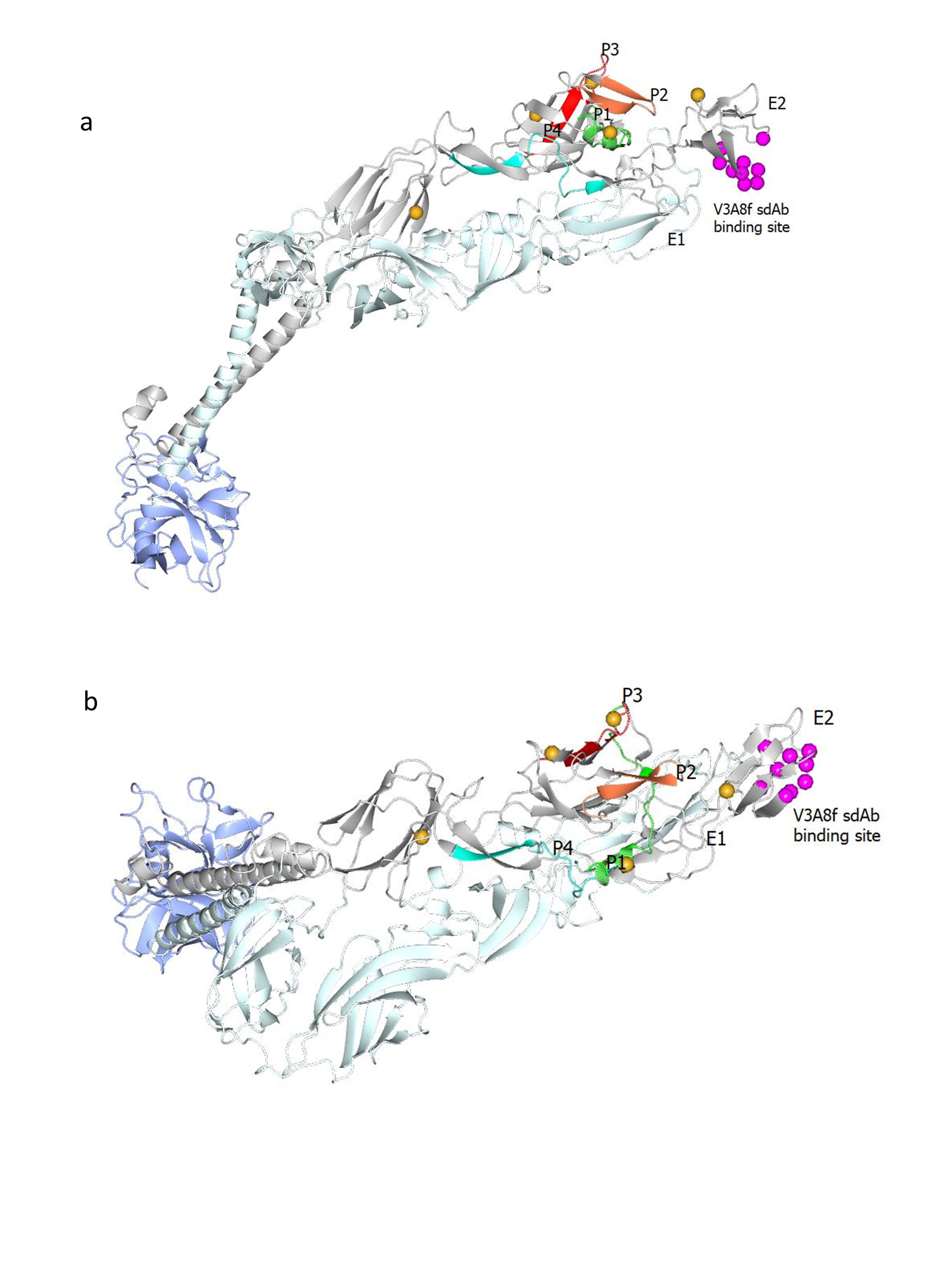

Supplement: Supplementary file 4 [file Image4.tif]
